# Preventing thromboembolism—another benefit of ERAS

**DOI:** 10.1093/bjsopen/zraf035

**Published:** 2025-04-09

**Authors:** David A Watters

**Affiliations:** School of Medicine and Health Sciences, Deakin University, Geelong, Victoria, Australia; Department of Surgery, University Hospital Geelong, Geelong, Victoria, Australia

Enhanced recovery after surgery (ERAS) was first introduced by Professor Henrik Kehlet in Denmark in 1997. ERAS bundles of care have since been developed for most major procedures. An umbrella review of meta-analyses performed by Zhang and colleagues in 2020 reported that, after 20 years, compliance with ERAS is associated with an average reduction in length of stay of 2.3 days^1^. This reduction is the product of both a faster recovery and fewer complications.

Despite variability in the economic methodology, the return on investment through delivering an ERAS bundle is in the order of 3–4:1. This is realized through the above reduction in hospital length of stay, fewer complications, but also lower readmission rates, amounting to an average cost saving of €609 (95% c.i. €890 to €222) per patient^1^.

ERAS should now be regarded as ‘best care’—an expected standard. Health services and/or units conducting major surgery should have their performance assessed against the delivery and outcomes of appropriate ERAS bundles. When measuring outcomes, particularly for complications such as venous thromboembolism (VTE), it is important that health data systems capture unplanned readmissions to any hospital, as up to half of VTEs occur after discharge and often present elsewhere.

Kirstin Black *et al.* report that ERAS compliance significantly correlates with a reduced incidence of VTE within 30 days, whereas VTE was associated with increased hospital costs of some €4100 per patient^2^. The study was substantial, reporting over 8000 operations in seven specialties from nine hospitals in Alberta, Canada.

Their findings confirm those of other smaller studies. Zhang *et al.* reported a series of 1143 hepatic resections for cancer with those treated under ERAS protocols enjoying a lower incidence of VTE, postoperative bleeding, and bile leak, but also shorter lengths of stay, reduced readmissions, and better long-term survival^3^. A smaller randomized study of 125 lung cancer patients reported a lower incidence of VTE (1.5% *versus* 11.7%) with lower D-Dimer levels on days 3 and 5 after surgery in those receiving the ERAS bundle^4^.

In Denmark, a country long associated with ERAS, Baastrup *et al.* reported on 1806 patients undergoing curative colorectal cancer surgery within an ERAS programme, with only 3 VTEs within 60 days^5^. All patients received thromboprophylaxis during their hospital admission, but only higher-risk patients received extended prophylaxis. Although this study was a retrospective cohort, its remarkably low incidence of VTE may well represent a ‘real world’ of ‘best care’ in which complications can be greatly reduced in a system providing ERAS as standard perioperative practice.

Research on ERAS has been beset by a multiplicity of procedures and conditions, combined with a certain amount of bundle creep. ERAS plus extends its reach to include surgery schools, preoperative optimization for co-morbidities, and prehabilitation. Although these all undoubtedly improve perioperative care and are within the remit of the ERAS Society’s mission, it does make it harder to prove which elements of the bundle are critical to the particular outcome(s) being measured.

In reducing the incidence of VTE, the Alberta study only reported statistical significance for early removal of urinary catheters and intravenous drips, compliance with prolonged preoperative sedation guidelines, and early postoperative mobilization^2^. However, ERAS protocols have multiple other components that likely combine to exert an overall effect. Certainly, overall bundle compliance above 70% was strongly associated with a reduced incidence of VTE^2^.

There is growing concern that too many elements are being added to some ERAS bundles, hindering implementation and compliance. The recent introduction of the simpler *Drinking, Eating, Mobilizing* (DrEaMing) may prove to be as effective. Henrik Kehlet warns DrEaMing is a wakeup call for ERAS^6^. At the very least, it avoids dehydration, promotes early eating (soft diet) and return of gastrointestinal function, and ensures early mobilization. However, comparative studies with ERAS bundles have not yet been done and so, for now, ERAS remains the gold standard.
